# Health aid displacement during a decade of conflict (2011–19) in Syria: an exploratory analysis

**DOI:** 10.1186/s12889-023-16428-7

**Published:** 2023-08-17

**Authors:** Munzer Alkhalil, Maher Alaref, Abdulkarim Ekzayez, Hala Mkhallalati, Nassim El Achi, Zedoun Alzoubi, Fouad Fouad, Muhammed Mansur Alatraş, Abdulhakim Ramadan, Sumit Mazumdar, Josephine Borghi, Preeti Patel

**Affiliations:** 1 Research for Health System Strengthening in Northern Syria (R4HSSS), Union for Medical and Relief Organizations, Gaziantep, Turkey; 2 Syria Public Health Network, London, UK; 3 LSE IDEAS Conflict and Civicness Research Group, London School of Economics and Political Science, London, UK; 4 Nottingham, UK; 5 Strategic Research Center SRC, Gaziantep, Turkey; 6 The Centre for Conflict & Health Research (CCHR), Research for Health System Strengthening in Northern Syria (R4HSSS), King’s College London, London, UK; 7 Syria Research Group (SyRG), Co-Hosted By the London School of Hygiene and Tropical Medicine and Saw Swee Hock School of Public Health, London, UK; 8 MEHAD, Paris, France; 9 Faculty of Health Sciences, American University of Beirut, Beirut, Lebanon; 10 Health Information System Unit, Gaziantep, Turkey; 11 Centre for Health Economics, University of York, York, UK; 12 Department of Global Health and Development, London School of Hygiene and Tropical Medicine, London, UK

**Keywords:** Health aid displacement, Fungibility, Syrian crisis, Conflict, IDPs, Humanitarian aid, Health aid, Development

## Abstract

**Background:**

Syria has been in continuous conflict since 2011, resulting in more than 874,000 deaths and 13.7 million internally displaced people (IDPs) and refugees. The health and humanitarian sectors have been severely affected by the protracted, complex conflict and have relied heavily on donor aid in the last decade. This study examines the extent and implications of health aid displacement in Syria during acute humanitarian health crises from 2011 to 2019.

**Methods:**

We conducted a trend analysis on data related to humanitarian and health aid for Syria between 2011 and 2019 from the OECD’s Creditor Reporting System. We linked the data obtained for health aid displacement to four key dimensions of the Syrian conflict. The data were compared with other fragile states. We conducted a workshop in Turkey and key informants with experts, policy makers and aid practitioners involved in the humanitarian and health response in Syria between August and October 2021 to corroborate the quantitative data obtained by analysing aid repository data.

**Results:**

The findings suggest that there was health aid displacement in Syria during key periods of crisis by a few key donors, such as the EU, Germany, Norway and Canada supporting responses to certain humanitarian crises. However, considering that the value of humanitarian aid is 50 times that of health aid, this displacement cannot be considered as critical. Also, there was insufficient evidence of health displacement across all donors.

The results also showed that the value of health aid as a proportion of aggregate health and humanitarian aid is only 2% in Syria, compared to 22% for the combined average of fragile states, which further indicates the predominance of humanitarian aid over health aid in the Syrian crisis context.

**Conclusion:**

This study highlights that in very complex conflict-affected contexts such as Syria, it is difficult to suggest the use of health aid displacement as an effective tool for aid-effectiveness for donors as it does not reflect domestic needs and priorities. Yet there seems to be evidence of slight displacement for individual donors. However, we can suggest that donors vastly prefer to focus their investment in the humanitarian sector rather than the health sector in conflict-affected areas. There is an urgent need to increase donors’ focus on Syria’s health development aid and adopt the humanitarian-development-peace nexus to improve aid effectiveness that aligns with the increasing health needs of local communities, including IDPs, in this protracted conflict.

## Key messages

• Health aid displacement is not an effective tool for aid-effectiveness used by donors in Syria.

• Some health aid displacement is evident for some leading donors such as the EU, Germany, Norway and Canada.

• Donors prefer to fund humanitarian activities with humanitarian aid being 50 fold more than development health aid; this is largely because humanitarian projects are easier to implement with sanctions and highly dynamic borders.

• There is an urgent need to adopt the humanitarian-development-peace nexus, considering the localization agenda. This approach can bridge the gap between the humanitarian and development phases, thereby increasing aid effectiveness.

## Background

The unprecedented number of protracted humanitarian emergencies in the last decade, including armed conflicts, natural disasters, political violence, human rights violations, climate change and COVID-19, have contributed to the greatest humanitarian challenges since World War II, leading to more than 100 million forcibly displaced persons, including refugees and internally displaced persons (IDPs) in May 2022 [1].

Many conflict and crisis-affected countries depend on donor aid (both humanitarian and development), particularly during times of crisis. However, it is unclear where humanitarian aid is additional or displaces other aid and whether crises affect the quality of aid. Also, it is unclear how donors used needs’ assessment data for decisions to allocate funds [2]. Reallocation of domestic health funds due to receiving donor health aid by the recipient government to match other priorities is known as health aid fungibility [3, 4], which has been long recognized with many studies showing donor aid is fungible in certain countries and sectors. For example, foreign aid is fungible in health, education, and agriculture, partially fungible in energy, but non-fungible in transport and communication [5]. Lu et al. found that between 1995 and 2006, there had been a decrease of almost US$0.43 in domestic health spending for each extra dollar of development assistance for health (DAH) [6]; so that health aid has a negative impact on domestic spending on health.

In comparison, very few studies have considered health aid displacement at the donor level, which refers to shifting funding from the health sector to other sectors, such as the humanitarian sector during crises to reflect the humanitarian needs and/or shifting priorities of donors; this displacement does not always consider domestic health needs during decision-making [7]. The limited existing evidence suggests that the flow of humanitarian aid during humanitarian crises was not at the cost of health aid. For instance, in Sierra Leone, South Sudan and Lebanon there was no evidence of health aid displacement during times of crisis [7–9].

However, there are no studies on health aid displacement in conflict settings in the MENA region. Syria offers a unique example of a protracted conflict for more than a decade, which has left a humanitarian crisis described as the worst of the twenty-first century [10]. With around 60% of Syrians (13.7 million) currently internally displaced or living as refugees [11]. Over 98% of individuals in Syria live in extreme poverty, living on less than $1.90 per person a day according to the Humanitarian Needs Assessment in September 2021 [12].

The main objectives of this paper are to analyse trends in humanitarian and health aid in Syria between 2011 and 2019 and their alignment with needs in terms of key dimensions of the Syrian conflict. We also examine whether there is evidence of health aid displacement across all donors, assess whether and how this differs between donors, and compare the volume of health and humanitarian aid in Syria to other fragile states.

## Study setting

**Fig. 1:**
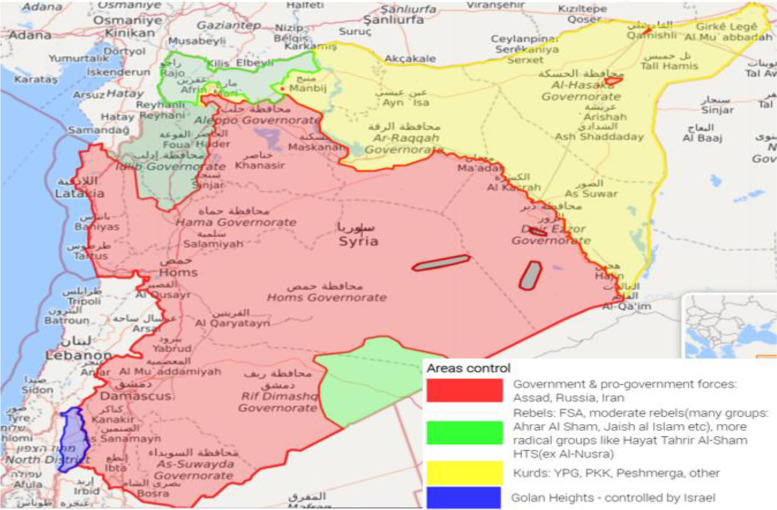
Areas of control in Syria as of Jan 2019—Source: Live Universal Awareness Map (Liveuamap)

Syria has been in a state of continuous conflict since 2011, resulting in more than 874,000 – directly and indirectly deaths [13, 14], The areas of military influence have changed dramatically in Syria between 2011 and 2019. The Syrian government control is limited to the red area in 2019 (Fig. 1) and has contested power with four de facto local governments that arose during different times [15–22].

In 2014, UN Security Council Resolution 2165 was issued to allow donors to provide humanitarian aid through four border crossings: Bab al-Salam and Bab al-Hawa on the Syrian-Turkish border, Al-Yarubiyah on the Syrian-Iraqi border and Al-Ramtha on the Syrian-Jordanian border, which were all not under the control of the Syrian government. This Resolution is to support areas outside the control of the Syrian government and does therefore not require its approval [23]. At the same time, humanitarian aid continued to flow to the Syrian Governmental-held areas (GoHA) through many humanitarian agencies in collaboration with the Syrian Regime [24]. After that three of these border crossings were closed in 2020 due to further UN Security Council Resolutions, and only the Bab al-Hawa crossing is maintained thus far.

### The humanitarian funding landscape

The ten most significant humanitarian donors according to OCHA in 2019 (the last year of our study) were: the USA, Germany, UK, EU, Canada, Norway, Denmark, Japan, France, and Sweden, respectively [25]. The sectors which received most of the humanitarian funding in 2019 were food security (not specified), health, multiple shared sectors, education, water sanitation and hygiene, emergency shelter/non-food items (NFI), and protection, respectively [25].

The humanitarian clustering model was established in Turkey for the humanitarian response in Syria across border in 2014 [26]. It is led by the Office for the Coordination of Humanitarian Affairs (OCHA), which is responsible for leading the Humanitarian Response Plans (HRPs) and Humanitarian Needs Overviews (HNOs) and documenting the development process [27]. Humanitarian intervention was carried out by the health cluster mechanisms through three leading platforms: Syria hub, Turkey Cross-border and Jordan Cross-border, the three platforms working together through the Whole of Syria approach (WoS) established in 2015 [28].

Moreover, another humanitarian hub emerged inside Syria in Al Hasaka governorate in 2017, coordinated by the North East Syria (NES) NGOs Forum. It manages several technical working groups and has loose links with the WoS approach in Amman, Jordan.

## Methods

### Study design

This is a mixed methods country case study that tracks aid trends and allocation from donor countries and organisations in response to the humanitarian crises in Syria. It is then combined with follow-up qualitative interviews with an expert panel of key informants and stakeholders in the humanitarian and health sectors in Syria to complete and corroborate results obtained by analysing aid repository data.

### Quantitative data

The research team charted the crisis timeline based on four quantitative indicators after excluding many others due to their limitations in the Syrian context. These indicators were then tested in the expert panel to see if they were suitable or if they required adaptation. The relevant humanitarian indicators to explain key dimensions of the Syrian conflict include: 1) the number of IDPs; 2) the number of people in need of humanitarian assistance; 3) the number or frequency of internal movements (displacements) due to the conflict and violence only. As Syria did not witness significant natural disasters during this period, the number of internal movements due to natural disasters was at the most 2,300 until the beginning of 2017. It then increased to 27,000 in 2018 and dropped to 17,000 in 2019 [29]. 4) the decline in Syria’s population between 2011 and 2019. Although there are other important indicators that can trace humanitarian flow and conflict intensity, such as the number of civilian casualties, data are very scarce on such indicators.

For this study, we could not include casualty figures as an important indicator because when compared to refugee data, casualty data is of a lesser standard of validity for the period. The United Nations stopped counting fatalities in the conflict by January 2014, citing the difficulty in accurately recording the constantly increasing toll [30].

After expanded discussion with the experts in the panel, we concluded that, although there were limitations associated with each indicator individually, quantitatively, reading these four indicators together would best express the crisis timeline in Syria.

#### Data sources

Data on internal movements and IDPs were collected from the Internal Displacement Monitoring Centre (IDMC), which is a comprehensive source of data and analysis of internal displacement worldwide. The Centre was established as part of the Norwegian Refugee Council (NRC) in 1998 [29]. And data on the Syrian population were collected from United Nations – Department of Economic and Social Affairs / Population Division. Data on people in need of humanitarian assistant were collected from Syria Humanitarian Needs Overviews, Syrian Arab Republic Humanitarian Assistance Response Plans, Strategic Response Plans, and Syrian Arab Republic Humanitarian Response Plans issued by OCHA over the study period.

Data on humanitarian and health aid for Syria were collected from the OECD's Creditor Reporting System (CRS) [31]. Despite some limitations of the CRS, this database is the most comprehensive one for tracking health and humanitarian aid for conflict-affected countries; it enables analysis of different aid activities, multilateral and philanthropic donors, country donors and recipients, purpose, policies, and over years [7, 32]. As per our knowledge, we did not find any other comprehensive sources that track aid in Syria.

The data, in the CRS, are reported by 42 multilateral donors (i.e., multilateral institutions such as UN agencies); 49 bilateral donors (i.e., country), and 36 private donors (i.e., entities such as the Bill and Melinda Gates Foundation).

The CRS provides financial data for 2002 -2021 with almost 200,000 – 300,000 data entries per year. Furthermore, it covers the specific economic or social programs that the aid seeks to support in a recipient country and classifies them into sectors. In addition, some contributions are not subject to sector-specific allocations and are reported as non-sector allocable aid [33]. Donors report which country is receiving aid and the purpose of aid. In addition, some descriptive information about the projects is also provided [34].

The CRS data used in this study are based on the 31 April 2021 update [31] and were downloaded to Excel sheets on 15 August 2021. The DAC and CRS list of codes were updated on 24 April 2021 [35].

The scope of the study is the whole of Syria whether the government- controlled area or the non-government-controlled territories.

#### Quantitative variables

Based on the databases mentioned earlier, we identified several variables representing crisis timelines, health aid and humanitarian aid. Table 1 provides a summary of the variables used in the quantitative analysis:

**Table 1:** List of variables included in the quantitative analysis

Domain	Variable	Source	Comments
Donors’ disbursements	Health aid	CRS	This variable was a sum of the following variables^a^: 1- Health General (121) 2- Basic Health (122) 3- Non-communicable diseases (123) 4- Population Policies/Programmes & Reproductive Health (130)
	Humanitarian aid	CRS	This variable was a sum of the following variables^b^: 1- Emergency response (720) 2- Reconstruction, relief, and rehabilitation (730) 3- Disaster prevention and preparedness (740)
Crisis Timeline	Number of IDPs	IDMC	Refers to people who are forced to leave their homes but have not crossed their national borders to find safety, unlike refugees, who have settled in or left for areas outside the national border [36].
	Number of people in need of humanitarian assistance	OCHA	Humanitarian aid programmes focus on saving lives in the short term [37],
	Frequency of internal movements due to the conflict and violence only	IDMC	Conflict, violence, and disasters, including floods, storms and droughts, force many people who have already been displaced to flee again; if the same person moves four times a year, four moves are recorded [38].
	Decline in Syria’s population	UN	This variable was extracted from United Nations – Department of Economic and Social Affairs / Population Division

^a^The CRS has “purpose codes” [33]; health aid was outlined by DAC 5 CODE 120: I.2. to 130: I.3. which includes Health General (121), Basic Health (122), Non-communicable Diseases (123), and Population Policies/Programmes & Reproductive Health (130). Therefore, health aid represents only non-humanitarian health, so there is no duplication with humanitarian aid

^b^Humanitarian aid was outlined by DAC 5 CODE with 700: III, which includes Emergency Response (720), Reconstruction, Relief, and Rehabilitation (730), and Disaster Prevention and Preparedness (740). In these subcategories, several health-related aspects are included within emergencies and humanitarian aspects. However, they do not intersect with non-humanitarian health subcategories in the health sector [39]

#### Quantitative data analysis

We collected information about the Syrian conflict's key dimensions from various sources annually between 2011 and 2019. Then we applied trend analysis techniques to trace their trajectories, which allowed us to comprehend the interrelations among them. Simultaneously, we explored the connections between the crisis timeline and the fluctuations in health and humanitarian aid trends.

We gathered financial data from the CRS and performed a trend analysis using Excel from 2011, the year the protests began in Syria, until 2019, the last year with available data at the CRS database at the moment of downloading the data. In this analysis, our definition of aid includes “Official Development Assistance”: “ODA grants” and “ODA loans” and “Private Development Finance” from the Bill & Melinda Gates Foundation (BMGF). In addition, aid excludes “Equity Investment” and “Other Official Flows” [7, 31, 40]. This combination is consistent with other recent analyses [7]. Data on regional and non-country-specific aid was not included in this analysis, and the focus was solely on aid flow to the Syrian territories.

We extracted data on gross disbursements rather than commitments because we were looking for “the actual international transfer of financial resources, or goods or services valued at the cost to the donor” [41]. To analyse aid trends over this long timeframe, we relied on constant 2019 US dollars rather than the current value to account for fluctuations in exchange rates and inflation. The Development Assistance Committee (DAC) deflator converts the amounts back to the value they held in a specific year. This means the expression of flows to multilateral donors and recipient countries is in terms of the purchasing power of the US dollar in each year of the study period [42, 43]. The aid database includes the bilateral ODA of the DAC members and excludes their contributions to the regular budgets of multilateral institutions when accounting for bilateral aid [42].

We considered that a decrease in health aid while an increase in humanitarian aid in a given year as a sign of health aid displacement.

### Qualitative data

#### Data sources

We also conducted semi-structured interviews by constituting a panel of humanitarian sector experts, humanitarian practitioners, and public sector officials; this was a crucial step, especially in this setting where quantitative data alone is often not an entirely accurate reflection of what is happening on the ground.

We used purposive sampling followed by snowballing sampling approaches to identify the participants. The research team invited 31 humanitarian workers in senior positions from health NGOs and INGOs, local authorities, technical entities, and the Turkey Cross-border health cluster for Syria’s response to an Expert Panel in Turkey – Mersin in August 2021. Twenty-five out of 31 accepted and attended the panel, 88% of the participants were from a health and medical background with vast experience in humanitarian and health programs. The discussions were conducted in Arabic with 3 research assistants writing notes as the participants did not agree on recording the session.

We also followed up findings from the Expert Panel with four key informants interviews (KIIs) with representatives of the four leading donors in September–October 2021 to understand the key stakeholders’ perspectives. The feedback from these key informants aligned strongly with the areas discussed in the Expert Panel. The KIIs were conducted in English and recorded. They were later transcribed and anonymized using a unique identifier for each participant. Following a thematic analysis approach, data from the Expert Panel and interviews were extracted and categorised into different themes.

#### Qualitative data analysis

For qualitative data we used a thematic analysis approach, data from the Expert Panel and interviews were extracted and categorised into different themes. The qualitative approach was aimed at completing, interpreting, and understanding the quantitative data-related results, comprehensiveness of the aid databases, and scope of the humanitarian and health interventions (emergency and/or development).

## Results

### Key dimensions of the Syrian conflict

**Fig. 2:**
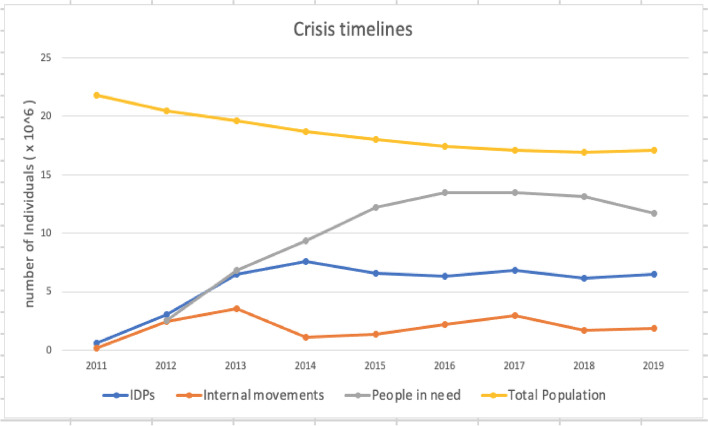
Crisis Timeline in Syria, 2011–2019 [38, 44–52]

It can be seen from Fig. 2 that the population of Syria is constantly decreasing, as the number dropped by about 4.7 million over the study period. The numbers of IDPs steadily rose until a peak in 2014 and then witnessed other increases in 2017 and 2019.

The volume of internal movements peaked in 2013 and in 2017. Finally, the number of people in need peaked in 2016 and 2017 with 13.5 million people in both years.

The four indicators reflect the severity of the humanitarian crises which showed an increase in the years 2013, 2014, 2016, 2017, and 2019. However, it seems that the year 2017 was worse due to the length of the crisis and the large number of violent incidents that occurred in 2016. A total of 338 attacks on health facilities were recorded across Syria, 38 attacks on education facilities and a quarter of the Syrian population lived in besieged or hard-to-reach areas [53]. Health aid displacement will be examined herein in response to the crisis timeline indicated in Fig. 2.

### Trends in aid flow concerning the key conflict and crises parameters

**Fig. 3:**
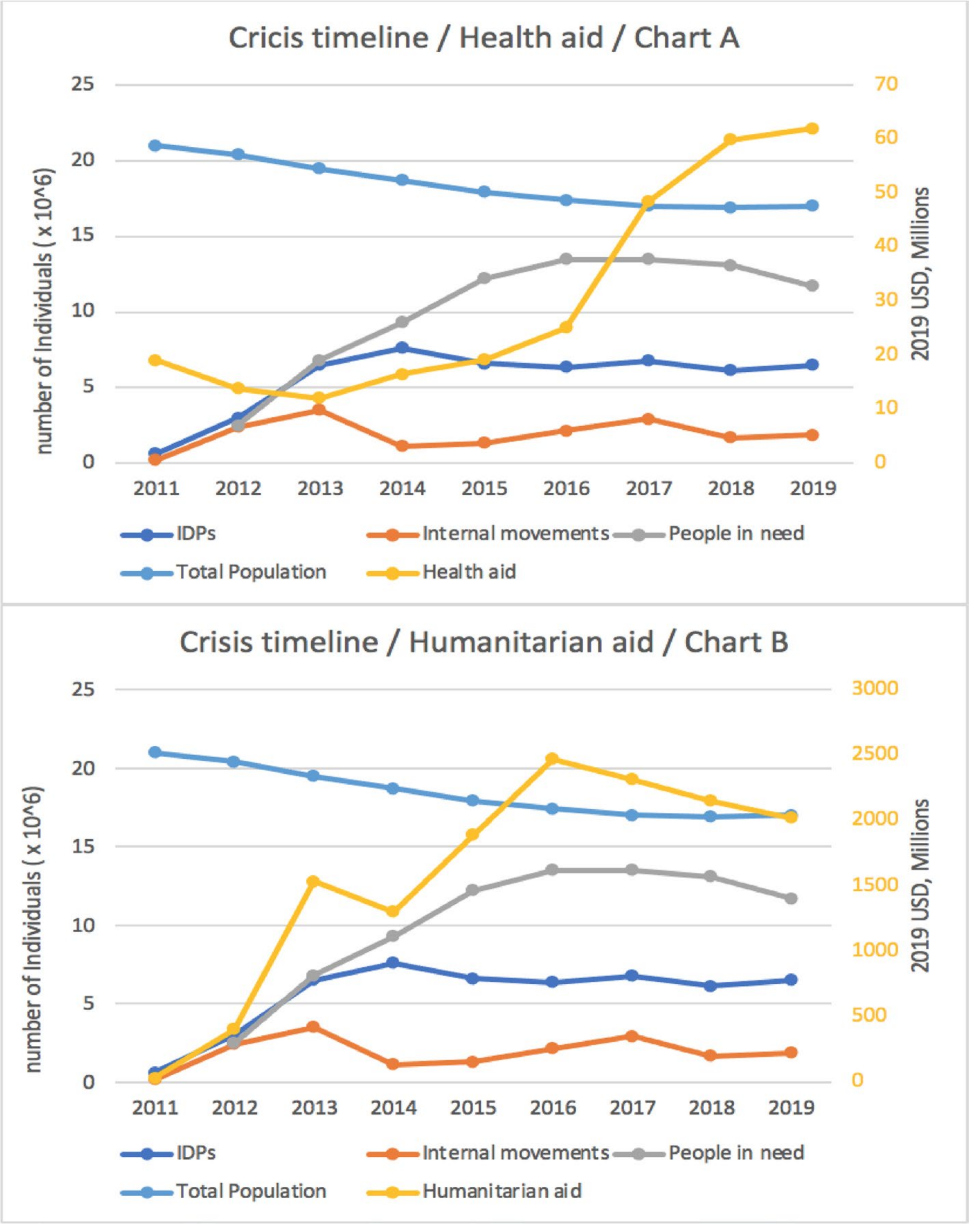
Humanitarian and health aid trends against the crisis timeline, 2011-2019

When considering aid trends against the crisis timeline, we notice a significant rise in health aid in 2017, which corresponds to the four indicators: the number of IDPs in Syria (6.78 m), number of internal movements (2.91 m), people in need (13.5 m), and the total population (17 m) (Fig. 3-Chart A). Notably, there were two peaks in humanitarian aid in 2013 and 2016 which corresponds to the number of internal movements index in 2013 (3.5 m) and the people in need index in 2016 (6.8 m). However, in 2014 there was no clear parallel with the humanitarian crisis expressed through the number of IDPs in Syria indicator, which peaked in 2014 at 7.6 m. Moreover, in 2017, which represents the peak of the humanitarian crisis in Syria, humanitarian funding decreased by 159 million compared to 2016 (Fig. 3 -Chart B).

With reference to Fig. 3, it seems that such indicators are not the major criteria for the allocation of humanitarian aid to Syria as none of them seem to strongly impact donor decisions for mobilization of funds.

Participants in the Expert Panel and interviews emphasized that there is no clear relationship between health and humanitarian funding allocations and the four indicators and that it is not clear that donors rely on need assessments before allocating funding. For example, the 2020 HRP, which was supposed to be released before the start of the year, was issued in December 2020, and the 2021 HRP was not released at the time of the workshop in Mersin in August 2021.

This raises an important question: Are HRPs developed after the projects are implemented?

### Health aid displacement

**Fig. 4:**
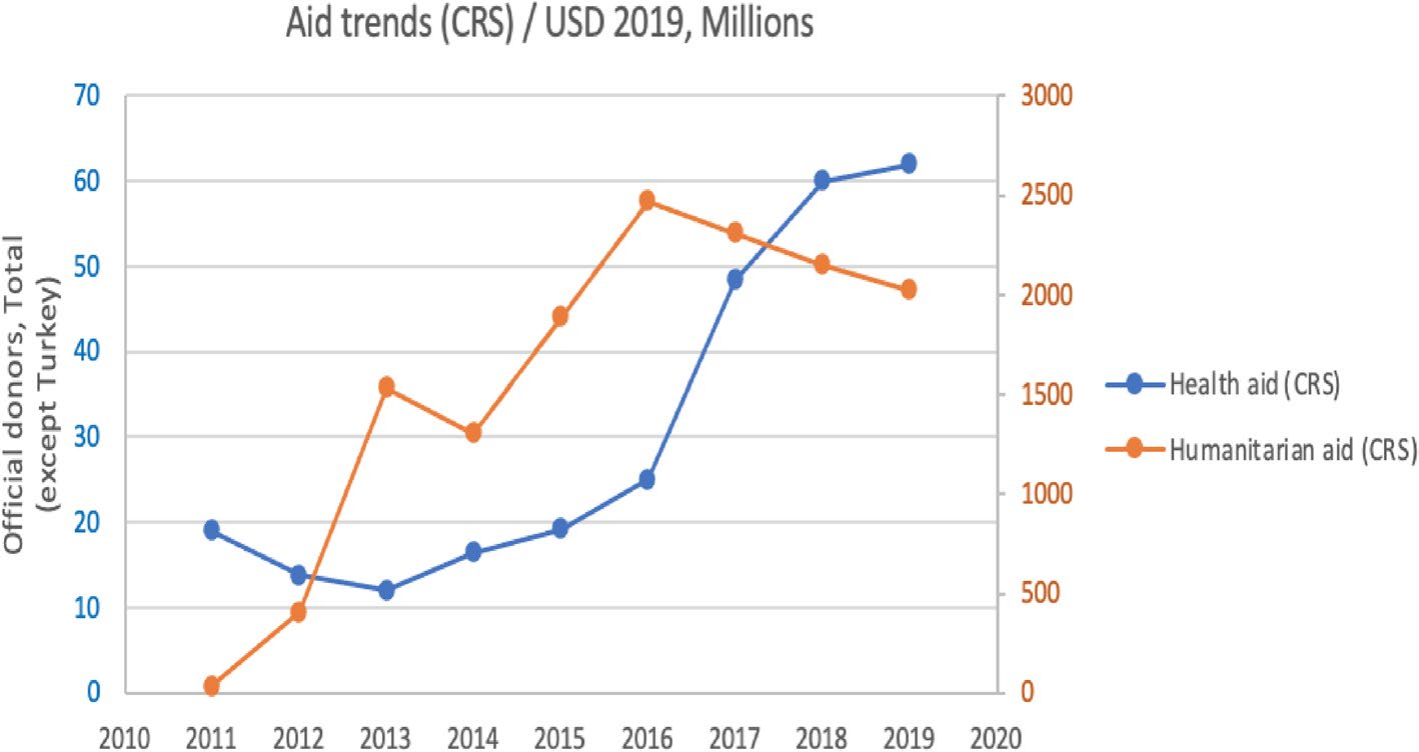
Humanitarian and health aid trends in Syria, 2011-2019

The trend in the volume of health aid vis-à-vis humanitarian aid evident from the CRS for all donors (except Turkey) indicates the latter to be about 50 times higher than health aid (Fig. 4). We excluded the Turkish donor from this analysis due to a classification error in their reporting on the CRS system; Turkish funds were spent on Syrian refugees in Turkey and not within Syrian territory [39].

It can be seen that health aid and humanitarian aid trends are, in fact, inversely correlated as health aid decreased by 7 $million, whereas humanitarian aid increased by 1.5 $billion between 2011 and 2013, and health aid increased when humanitarian aid decreased after 2016. However, the magnitude of financial flows is very different. The increase in humanitarian aid from 2011 to 2013 was far more significant in scale than the corresponding decrease in health aid, so this aid displacement is negligeable. Moreover, between 2013 and 2019, there was no health aid displacement at this aggregate level.

The Expert Panel and KIIs all stated that although CRS database is a great resource, they questioned the reliability of data presented in this work given funds from Gulf countries at the beginning of the crisis were not recorded, as well as other sources of private funding such as individual donations, therefore CRS misses an important source of funding. This finding is in accordance with the literature which also states that a major limitation in CRS is the omission of data from countries which do not report their aid disbursements to the CRS which includes China, Qatar, and Saudi Arabia [32, 54].

The participants also stressed the importance of excluding Turkey as a donor and that the amounts registered as humanitarian aid within Syria are, in fact, aid that was disbursed to Syrian refugees within Turkey, also emphasizing that there are not many precise details on how these amounts were spent.

### Health aid displacement for individual donors

**Fig. 5:**
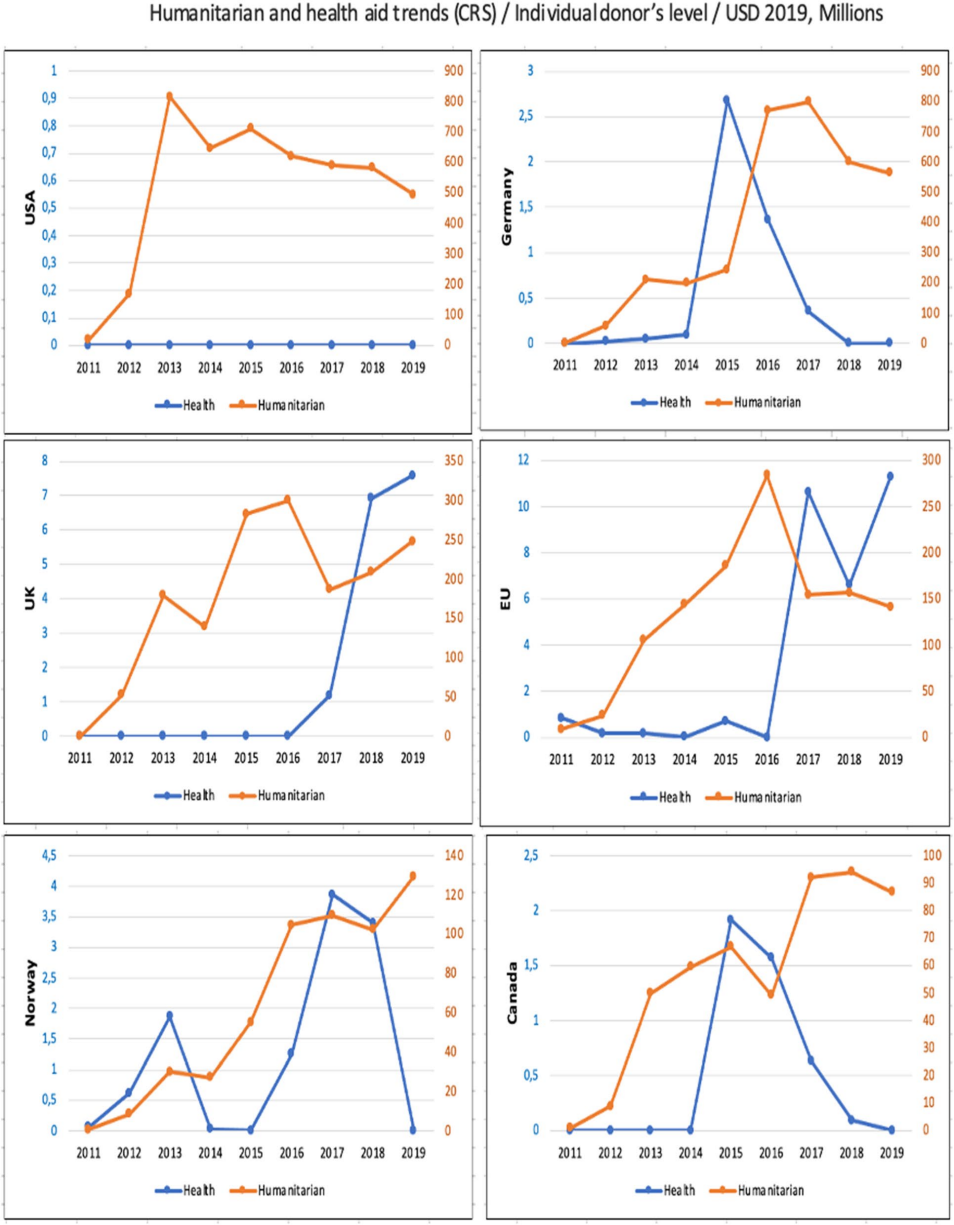
Aid trends at individual donor's level, 2011–2019

The following six charts show the health aid displacement at the level of the six largest donors in the health and humanitarian sectors in Syria between 2011 and 2019: the USA, Germany, the UK, EU, Norway, and Canada (Fig. 5).

The United States did not spend on health during the study period. Instead, it focused on humanitarian aid, so there is no displacement of health aid for this donor. For Germany, there was an apparent decrease in spending on health with $2.3 million per year between 2015 and 2017 and spending reached zero in 2018 and 2019; this was accompanied by an increase in spending on humanitarian aid with $545 million per year between 2015 and 2017, which, theoretically, could be considered a displacement of health aid.

The UK did not spend on health in the first six years, while its spending has increased steadily in the last three years; no evidence of health aid displacement from the UK.

In the first six years, European Union spending on health was close to zero, then increased in the following three years. In 2017 and 2019 there was a shift in EU investment in favour of health aid, while, in 2018, spending on health decreased by $4 million, and there was an increase in spending on humanitarian funding by approximately $2.3 million in the same year; this could be considered health aid displacement.

For Norway, in 2019, the decrease in health funding from $3.4 million to zero was accompanied by an increase in humanitarian funding by nearly $27 million, and this can be considered a health aid displacement.

Finally, for Canada, there was an apparent decrease in health funding after 2016 from almost $1.6 million per year until it reached 0 in 2019, and this was accompanied by an increase in humanitarian funding by almost $37.5 million per year during the same period, which can be considered health aid displacement.

A key takeaway is that although there is slight health aid displacement at the level of some donors in specific years especially after 2015 there is no consistent evidence of displacement over the study period. Also, by comparing the individual donors’ result with aggregate data in Fig. 3, the health aid displacement was at individual donors’ level for some years but was not accompanied by health aid displacement across donors.

### Comparison between health aid in Syria and other fragile states

**Fig. 6:**
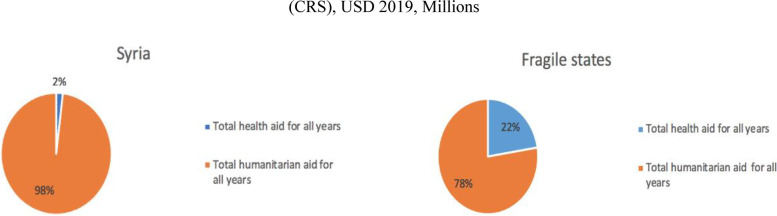
The percentage of health aid out of the humanitarian and health aid in Syria compared to other fragile states (CRS), USD 2019, Millions

Figure 6 shows that the proportion of health aid to the combined total of health and humanitarian aid in Syria is 2% compared to 22% in other highly fragile countries on average. This means that in the Syrian context, donors’ are prioritizing the humanitarian sector relative to health, and this was confirmed among our participants in the Expert Panel and KIIs.

The reason for humanitarian aid favouritism between 2011 and 2019 mentioned by participants in the Expert Panel and KIIs is the weakness of a governance structure in opposition-led areas such as north west Syria and north east Syria. This might discourage funding health programs as a development investment. So, donors prefer funding emergency health programs defined as humanitarian through UN agencies because it is less risky in terms of aid diversion and tends to be less politicised.

Also, the participants in the Expert Panel and KIIs pointed out that the multiplicity of governments, the different areas of military control, and the governance complexity make donors more inclined towards humanitarian aid rather than health aid of a developmental nature. The dramatic change in military influence over the last decade was accompanied by significant changes at the level of governance and the management of humanitarian and health aid.

In addition, the participants in the Expert Panel and KIIs mentioned the “blurred lines” between humanitarian and development health aid. Most of the participants confirmed that many humanitarian actors provide health services in a development sense, and that could be because many local NGOs are led and managed by individuals from a health and medical background. The definition of health programs in humanitarian emergency and development settings is confusing, and the boundaries between the two scopes are unclear. In the same course, the participants questioned the standardised methodology and definitions used to classify health programs implemented inside Syria by donors in CRS database, and whether the mix between humanitarian and developmental activities on the ground also affects the reporting and thus the reliability of the distinction between humanitarian and health aid in CRS database.

## Discussion

To the best of our knowledge, this is the first study that provides an analysis of health and humanitarian aid trends and explores health aid displacement in a conflict-affected setting in the MENA region. It covers the Syrian crisis from its start in 2011 until the year 2019, the latest available information on the CRS database at the downloading data moment; we wanted to look at all the available conflict years to study the trends.

We present a crisis timeline while considering different criteria -number of IDPs, total population, number of internal movements, and number of people in need. However, it seems that none of these indicators strongly influenced donor aid allocation in Syria. This is consistent with other literature which identifies alternative criteria that donor countries take in their decision- making process such as perceived risk of investment, colonial/post-colonial links, nationalist policies, domestic concerns, dynamics of a conflict [55, 56].

Although we observed reductions in health aid disbursements alongside increases in humanitarian aid among donors such as the EU, Germany, Norway and Canada, the scale of the increased humanitarian funding in all cases except the EU far exceeded that of reductions in health aid. This suggests that while health aid displacement may have occurred, most of the increase in humanitarian aid was additional, increasing the overall health and humanitarian aid envelope. In comparison, we observed in the literature a different pattern in other conflict settings such as South Sudan, Sierra Leone, and Lebanon where humanitarian aid was added to the health aid and did not replace it [7–9].

However, when comparing the ratio of humanitarian aid to health aid given to Syria to that of all other fragile states, there is a strong indication that donors prefer to fund humanitarian activities in Syria as the ratio is 2 to 98 in favor of humanitarian aid compared to 22 to 78 in the other fragile countries.

Humanitarian aid can include funding to the health sector, and sometimes donors label health aid as humanitarian aid in humanitarian crisis settings. According to OCHA, in 2019, 8,6% of Syria’s humanitarian aid was health-related [25]. This part includes, primarily, life-saving health aid and sometimes health aid of a developmental nature. A clear example of that, the German Donor / Federal Minister for Economic Cooperation and Development (BMZ) supported 8 health directorates in the area outside of the Syrian Regime’s control, which are health local authorities, between 2017 and 2019 through Deutsche Gesellschaft für Internationale Zusammenarbeit (GIZ) in a development sense [57]. The GIZ mandate is “sustainable development and international education work” [58]. We see in Fig. 5 that Germany’s health aid to Syria in 2018 and 2019 was zero. That means even strengthening health system activities through development entities have been provided under the humanitarian, not health, umbrella.

One would also argue that given the protracted nature of the Syrian conflict, humanitarian aid would somehow cross with development health aid given that most humanitarian agencies, donors and INGOs try to adopt the humanitarian-development-peace nexus [59] which calls for using a holistic approach where coherence among the development, humanitarian and peace-related actors, policies and operations should be ensured. These “blurred lines” between humanitarian and development aid have been mentioned in the literature with more people speak of a humanitarian development “continuum” [60–62]. However, major challenges in implementing the nexus in other conflict settings like Uganda [63].

Preference for humanitarian aid was confirmed by the participants in the Expert Panel and KIIs due to the governance challenges and donors’ desire to avoid dealing with the Syrian Regime largely due to its involvement in war crimes and crimes against humanity. This is consistent with the literature on health governance in opposition-held areas that shows that although there is a form of quasi-governance in both NWS and NES, donors are hesitant to support health directorates as they believe they lack capacity. Donors also prefer to work through NGOs partially due to concerns about treating opposition-controlled health authorities as political substitutes for the Syrian regime [64, 65]. Also, this can be because at the earlier stages of the Syrian crisis, many countries, including major donors, imposed unilateral sanctions [66–69] on the Assad regime which made it less likely for them to provide health aid. However, those sanctions were designed while ensuring that they would not affect humanitarian assistance [70], which made it much easier and more realistic to focus primarily on humanitarian aid.

The reason why the United States did not invest in health aid of a developmental nature is mostly its stance against the violations of the Syrian regime since the protests began. First, the US government imposed economic sanctions on officials in the Syrian regime less than a month after the protests began [71], then, followed by a series of sanctions against many officials, including the Syrian president [71], his wife [72], his foreign minister [73], and many businesspeople close to the Assad family [72, 73].

Despite its limited amounts, health aid by most donors seems to be decreasing annually, except for UK and EU donors, at a faster pace than that of humanitarian aid. However, this is not the case with the overall trends where health aid is slightly increasing annually, contrary to humanitarian aid which is decreasing.

This slight increase in “interest” in health aid by UK and EU may be primarily due to a major change in the political climate in Syria: the international community has partially resigned to the fact that the Assad regime is going to stay in power, so a political transition is unlikely. A few of the European embassies and Arab countries have reopened in Damascus with Assad’s diplomatic visits on the rise [74, 75].

Western countries are gradually easing restrictions on supporting and dealing with Syrian state institutions, so it is expected that development aid will increase in the coming years. Based on the Expert Panel and KIIs, this shift in the political environment would pose critical ethical challenges for the donors and the international community: How can donors support developmental health aid in light of a complex governmental reality in Syria? This is astounding given that the Syrian government has almost completely controlled state institutions for more than five decades.

The health cluster mechanism in Syria, operating under the WoS approach, primarily focused on providing life-saving services during the first decade of the Syrian crisis [76]. As a result, it did not significantly encourage donors to invest more in sustainable health interventions, including development health aid. This emphasis on immediate and critical healthcare needs reflects the humanitarian phase of the response. However, UN agencies have called recently for greater support and have taken steps toward increasing early recovery projects inside the country and thus re-adding development aid, including health aid, to major donors’ agendas. Indeed, in November 2021, the US Treasury Department’s Office of Foreign Assets Control (OFAC) announced its decision to expand authorizations for NGOs to engage in additional activities in Syria [77].

Estimates in this study may be incomplete as some donors, such as China, Qatar, and Saudi Arabia, also, other sources of private funding, do not report their aid disbursements to the CRS [32, 54, 78, 79]. And some donors, such as Turkey, do not report their aid with complete accuracy [39].

The population indicator may not be an ideal indicator of the crisis timeline, but it has been used due to the lack of regular updates on the number of refugees, deaths, and new births.

Although the number of IDPs and internal movements are suitable indicators for expressing the fundamental crises, they cannot represent the entirety of all the crises in the Syrian context, as many areas have witnessed significant humanitarian crises due to the military siege. For example, a high-level UN investigation reported in 2018 that the more than five-year siege of Eastern Ghouta in a war-torn country is “barbaric and medieval” [80]. However, this disaster did not significantly affect the number of IDPs and internal movements over the years because 400,000 people, including wounded people, were prevented from leaving their homes due to the military blockade [81]. In addition, there are significant numbers of people who migrated outside Syria. According to the UNHCR, the number of the Syrian refugees worldwide was 6.8 million in 2022 [11]. This is in addition to more than 874,000 deaths since 2011 due to the conflict [82].

## Recommendations

We highlighted above several challenges of aid that are faced in Syria, a highly complex conflict setting with multiple local, regional, and international players along with state and non-state actors and combatants. A few recommendations can be made which would help in improving aid at different levels: the OECD should do more to ensure having more inclusive databases that go beyond the current donors reported to CRS, such as including China and other so-called “emerging” donors that do not share comprehensive information about their aid funds. And there should be a clear distinction between humanitarian and health aid categories at the levels of the UN, OECD, donors, and recipient governments. Also, the OECD Secretariat should ensure quality and comparability by regularly reviewing donor input.

UN-OCHA should encourage donors to invest more in the health sector in Syria. It can prepare an annual response/development plan independent of the humanitarian response plan with more involvement for the local governance bodies in different areas.

In the humanitarian phase of the conflict in Syria, there was understandably limited engagement from humanitarian donors and organisations with local governments, considering the involvement of these governments in conflict atrocities. However, as we transition into the early recovery phase it becomes crucial to employ the humanitarian-development-peace nexus. This approach encourages simultaneous and synergistic efforts on humanitarian aid, sustainable development, and peacebuilding. The nexus becomes especially significant with the increased need to implement the localization agenda for sustainable humanitarian interventions and to pave the way for development, demanding more engagement with local actors.

It is essential to recognise that Syria now has new “de-facto borders” with different areas of control, a reality that has already been established by the 12 years of conflict. Therefore, a single approach to early recovery for the whole country is neither feasible nor best practice. The UN agencies and donors should prioritise efforts in enhancing health governance and development activities within each of the three main areas of control, including NWS and NES, to bridge the gap between the humanitarian and development phases.

To initiate development activities, it is important to assess the capability of each area and foster positive competition among them. At the central levels (NWS, NES and GoHA) donors should exert diplomatic pressure and engage in technical negotiations with the various central and local governments. This engagement facilitates meaningful involvement at the community and district levels, while harmonizing activities at the national level.

The lack of political engagement with governments involved in the conflict has provided them with opportunities to exploit humanitarian responses, evade public accountability, divert funds towards perpetuating violence and conflict, and exert control over aid distribution. The case of South Sudan serves as an example of these challenges [56].

Addressing these issues necessitates providing robust support to technical health bodies that play quasi-governmental or governmental roles at local and community levels based on human rights principles becomes imperative. This support can contribute to the formulation of comprehensive national needs strategies and plans, ultimately enhancing the effectiveness of health aid in Syria during the early recovery phase.

## Conclusion

In this study, we realized that in contexts as complicated as that of Syria, there was insufficient evidence of health displacement across donors. Yet there seems to be evidence of slight displacement for individual donors.

There is a strong indication that donors prefer to fund humanitarian activities, including health, in favor of humanitarian aid in Syria compared to other fragile countries. Especially that humanitarian aid was 50 folds more than development health aid, as humanitarian projects are easier to implement with sanctions and highly dynamic borders. UN agencies and INGOs should work more on providing more inclusive and better-defined aid reporting systems to ensure that all the received aid to a given country is recorded and that there is a clear distinction between humanitarian and development aid. This will provide more reliable evidence for policymakers to advocate for more development projects, especially in protracted conflicts such as Syria, where humanitarian projects cannot meet local needs.

The humanitarian-development-peace nexus should also be implemented to bridge the gap between the humanitarian and development phases. This can be achieved through leading technical negotiations by UN agencies and donors with the various central and local governments to facilitate meaningful engagement at the community and district levels. This approach helps to reduce the politicization of aid, empowers local communities, and enhances their ownership of development plans until a political solution matures in Syria.

The data used for quantitative analysis herein are based on donor reporting and donors' inputs without results and achievement-based reflection. And therefore, future work should focus on aid effectiveness in reference to the five goals of the 2005 Paris Declaration: Alignment, Harmony, Ownership, Results and Mutual Accountability.

## Data Availability

The datasets generated and analysed through the FGDs and KIIs are not publicly available as they contain information that could compromise research participant confidentiality. All other data sources used in this study were publicly accessible.

## Abbreviations

GoHA: Governmental-held Areas
IDPs: Internally Displaced People
OECD: Organisation for Economic Co-operation and Development
CRS: Creditor Reporting System
EU: European Union
COVID-19: Coronavirus Disease 2019
DAH: Development Assistance for Health
MENA: Middle East and North Africa
UN: United Nations
OCHA: Office for the Coordination of Humanitarian Affairs
USA: United States of America
UK: United Kingdom
NFI: Non-Food Items
HRPs: Humanitarian Response Plans
HNOs: Humanitarian Needs Overviews
WoS: Whole of Syria
NES: North East Syria
NGOs: Non-Governmental Organizations
IDMC: Internal Displacement Monitoring Centre
NRC: Norwegian Refugee Council
DAC: Development Assistance Committee
ODA: Official Development Assistance
BMGF: Bill & Melinda Gates Foundation
INGOs: International Non-Governmental Organizations
KIIs: Key Informants Interviews
BMZ: Federal Minister for Economic Cooperation and Development
HRPs: Humanitarian Response Plans
GIZ: Deutsche Gesellschaft für Internationale Zusammenarbeit
NWS: North West Syria
NES: North East Syria
OFAC: Office of Foreign Assets Control
UNHCR: United Nations High Commissioner for Refugees
